# Nomogram for preoperative estimation risk of lateral cervical lymph node metastasis in papillary thyroid carcinoma: a multicenter study

**DOI:** 10.1186/s40644-023-00568-5

**Published:** 2023-06-01

**Authors:** Jialin Zhu, Luchen Chang, Dai Li, Bing Yue, Xueqing Wei, Deyi Li, Xi Wei

**Affiliations:** 1 Department of Diagnostic and Therapeutic Ultrasonography, Tianjin Medical University Cancer Institute and Hospital, National Clinical Research Center for Cancer, Key laboratory of Cancer Prevention and Therapy, Tianjin’s Clinical Research Center for Cancer, Tianjin, 300060 China; 2 Department of Geriatrics, Tianjin Medical University General Hospital, Tianjin Geriatrics Institute, Tianjin Medical University General Hospital, Tianjin, 300060 China

**Keywords:** Nomogram, Lateral lymph node metastasis, Papillary thyroid carcinoma, Ultrasonography

## Abstract

**Background:**

Lateral lymph node metastasis (LLNM) is frequent in papillary thyroid carcinoma (PTC) and is associated with a poor prognosis. This study aimed to developed a clinical-ultrasound (Clin-US) nomogram to predict LLNM in patients with PTC.

**Methods:**

In total, 2612 PTC patients from two hospitals (H1: 1732 patients in the training cohort and 578 patients in the internal testing cohort; H2: 302 patients in the external testing cohort) were retrospectively enrolled. The associations between LLNM and preoperative clinical and sonographic characteristics were evaluated by the univariable and multivariable logistic regression analysis. The Clin-US nomogram was built basing on multivariate logistic regression analysis. The predicting performance of Clin-US nomogram was evaluated by calibration, discrimination and clinical usefulness.

**Results:**

The age, gender, maximum diameter of tumor (tumor size), tumor position, internal echo, microcalcification, vascularization, mulifocality, and ratio of abutment/perimeter (A/P) > 0.25 were independently associated with LLNM metastatic status. In the multivariate analysis, gender, tumor size, mulifocality, position, microcacification, and A/P > 0.25 were independent correlative factors. Comparing the Clin-US nomogram and US features, Clin-US nomogram had the highest AUC both in the training cohort and testing cohorts. The Clin‑US model revealed good discrimination between PTC with LLNM and without LLNM in the training cohort (AUC = 0.813), internal testing cohort (AUC = 0.815) and external testing cohort (AUC = 0.870).

**Conclusion:**

Our findings suggest that the ClinUS nomogram we newly developed can effectively predict LLNM in PTC patients and could help clinicians choose appropriate surgical procedures.

**Supplementary Information:**

The online version contains supplementary material available at 10.1186/s40644-023-00568-5.

## Background

Thyroid carcinoma is the most common malignant tumor of endocrine system, ranking 9th in incidence in 2020 [1]. Papillary thyroid carcinoma (PTC) is the commonest histological type of thyroid cancer. Studies have indicated that lateral lymph node metastasis (LLNM) occurs in 18-64% of PTC patients [2]. The presence of LLNM is a known prognostic factor for poor prognosis and high mortality after surgery in PTC [3].

Ultrasound (US) is the mainly method for assessing preoperative lymphatic status. However, the sensitivity of US diagnosis for cervical lymph node metastasis (LNM) is only 20-40% [4, 5]. It is reported that 18.6–64% of patients with PTC had occult LLNM with clinical negative (cN0) lateral neck [6]. Prophylactic lateral neck dissection (LND) is not suggested for lateral neck cN0 patients in majority of clinical guidelines, including American Thyroid Association (ATA) guidelines [7] and National Comprehensive Cancer Network [8]. It is recommended that therapeutic lateral neck dissection should be performed for PTC patients with metastatic lateral cervical lymphadenopathy confirmed by biopsy according to the 2015 ATA guidelines. The Japanese Society of Thyroid Surgeons and Japan Association of Endocrine Surgeons did not recommend prophylactic modified radical neck dissection as a routine method, which had not been proved to be beneficial [9]. Therefore, non-invasive assessment of lateral lymph node status in patients with PTC is of great value for clinical decision-making.

The nomogram is a kind of graphical calculation sliding rule to predict the probability of an event occurring, which has been widely used in the medical field in recent years. Combined with significant variables of regression analysis, nomogram has been extensively used in clinical practice because of its objectivity and convenience [10]. However, it is rarely used in the assessment of LLNM in PTC patients, especially in establishing nomogram based entirely on preoperative data.

In this study, we aimed to establish a preoperative estimating nomogram based on clinical and ultrasonic features (Clin-US nomogram) to predict the risk of LLNM in patients with PTC, and propose individualized treatment strategies to help clinicians make appropriate clinical decisions.

## Methods

### Patients and cohorts

This retrospective study was approved by the Ethics Committee of the Tianjin medical cancer institute and hospital, and the requirement for informed consent was waived. Patients were enrolled based on the following criteria: (1) Pathological examinations were performed to confirm PTC with/ without LLNM by surgery and fine needle aspiration cytology (FNAC). (2) Patients with clear US imaging of the thyroid nodules. (3) Patients with complete clinical information. (4) BRAF V600E analysis was necessary in hospital 1. Exclusion criteria were as follows: (1) Poor US imaging quality. (2) Cases with incomplete clinicopathological information. (3) Patients who had received preoperative therapy before image acquisition.

**Fig. 1:**
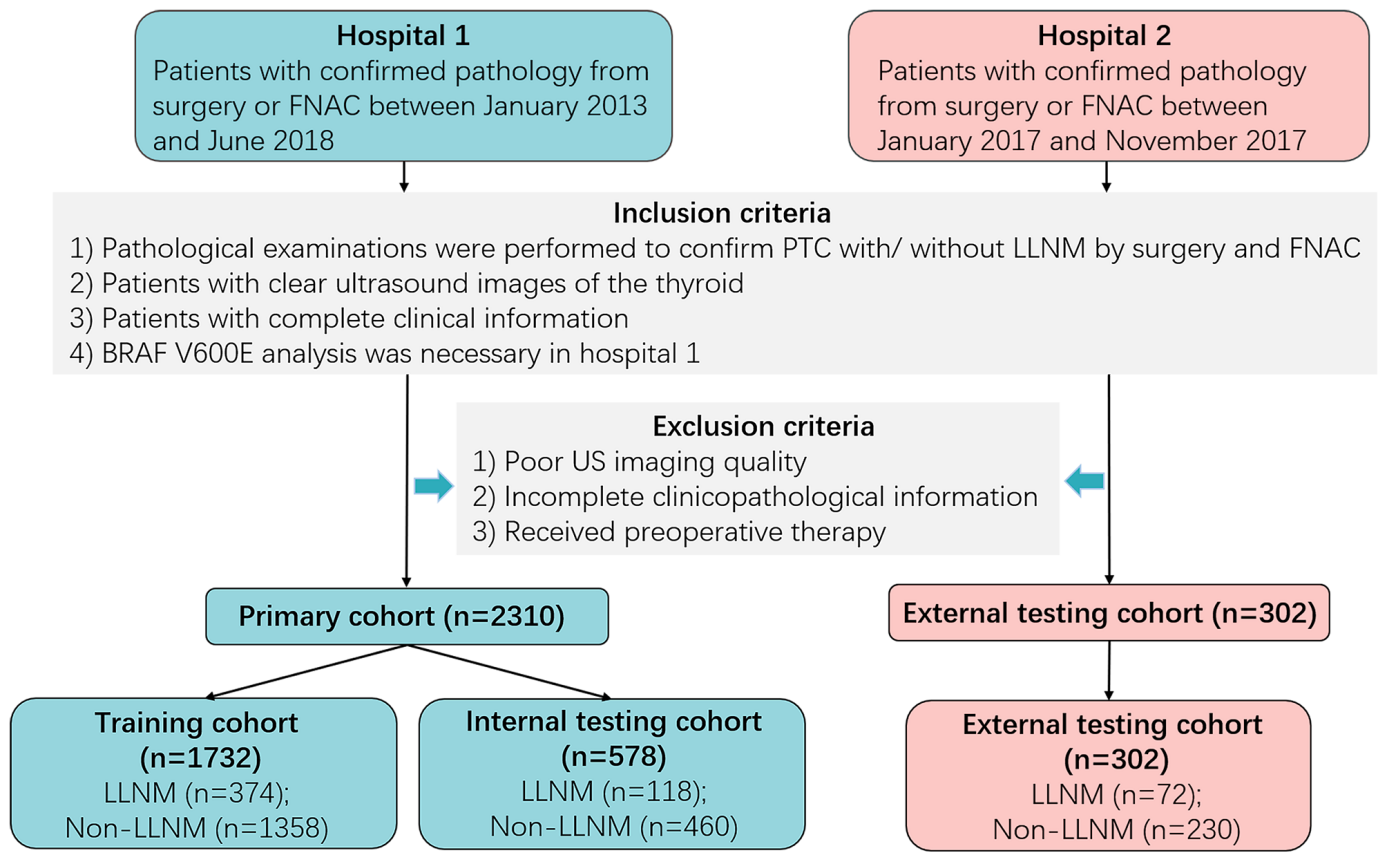
Flow chart of the patients enrolled in our study. Finally, 2612 PTC patients from two centers were reviewed

We retrospectively evaluated the patients who received thyroid surgery and histologically confirmed PTC in hospital 1 (Tianjin Medical University Cancer Institute and Hospital, Tianjin, China) between January 2013 to June 2018 and hospital 2 (Binzhou Medical University Hospital, Shandong, China) between January 2017 and November 2017. We analyzed routine clinical, US data and pathological results, and a total of 2612 patients with PTC were included in this study. In hospital 1, all patients (n = 2310) were randomly divided into a training cohort (n = 1732) and an internal testing cohort (n = 578) for developing and evaluating the nomogram. Patients in training cohort from hospital 1 were divided into a non‑LLNM group and a LLNM group according to the pathological results. Patients in hospital 2 were used as an external testing cohort (n = 302). Fig. 1 illustrates the flow chart of recruitment process of the final study patients.

### FNAC and surgical strategy

US-guided FNAC was performed by radiologists with more than 15 years of experience in thyroid FNAC with at least three repeated aspirations were performed in different directions for each nodule, using 22-gauge needles and preserving the remaining specimen in normal saline for BRAFV600E mutation analysis. According to US-guided FNAC, all patients enrolled in this study were confirmed as Bethesda Categories V or VI.

Cervical lymph nodes with spherical shape, normal echo of lymphatic hilum disappeared, cystic components, microcalcifications or peripheral vascularity were suspected to be metastatic [8, 11]. FNAC and thyroglobulin (TG) test were also conducted on the most suspicious lateral cervical lymph nodes before surgery to confirm the pathological diagnosis.

Total thyroidectomy or thyroid lobectomy were performed on all patients, along with central neck dissection (CND) according to the Chinese guidelines for diagnosis and treatment of differentiated thyroid carcinoma. According to the ATA [8] and Chinese guidelines, only patients with highly suspected metastatic lateral neck lymph nodes based on preoperative imaging data, FNAC and TG test, underwent LND, which comprised of removal of the lateral lymph nodes from level II to V, while preserving the internal jugular vein, spinal accessory nerve, or sternocleidomastoid muscle.

### Clinical data and ultrasound images

Clinical information, ultrasonic measurements and features were collected for data analysis. Clinical information included age and gender. US examination of PTC patients was operated by experienced radiologists with more than 8 years of experience in thyroid diagnosis. The US machine included Phillips EPIQ 5, IU 22, HD11, (Philips Healthcare, Eindhoven, The Netherlands), and Aplio 400, 500 (Toshiba Medical Systems, Tokyo, Japan) devices equipped with 5–12 MHz or a 4.8–11 MHz linear array probe.

The following characteristics were evaluated for all selected thyroid nodules: maximum diameter of tumor (tumor size), location, position, mulifocality, composition, echogenicity, margin, shape (A/T ≥ 1 or < 1), and microcalcification. Vascularization (blood flow) was classified according to the Adler grade of blood flow from 0 to 3 [12]. In addition, Hashimoto’s thyroiditis and the adjacent relationship with thyroid capsule were evaluated on the basis of US images. Hashimoto’s thyroiditis manifests as uneven echogenicity of the thyroid parenchyma on ultrasonography, with a few or multiple lamellar hypoechoic areas showing grid-like changes. The abutment was defined as the edge of the thyroid nodule contacting with the thyroid capsule. The measurement of abutment of the perimeter (abutment/perimeter, A/P) in a thyroid lesion was calculated by the average ratio (1/2) on the transverse + longitudinal section of a nodule. Based on our previous study, we calculated the 1/4 (25%) perimeter of the thyroid lesion as the cutoff value[13].

### Feature selection and clin-US nomogram development

**Fig. 2:**
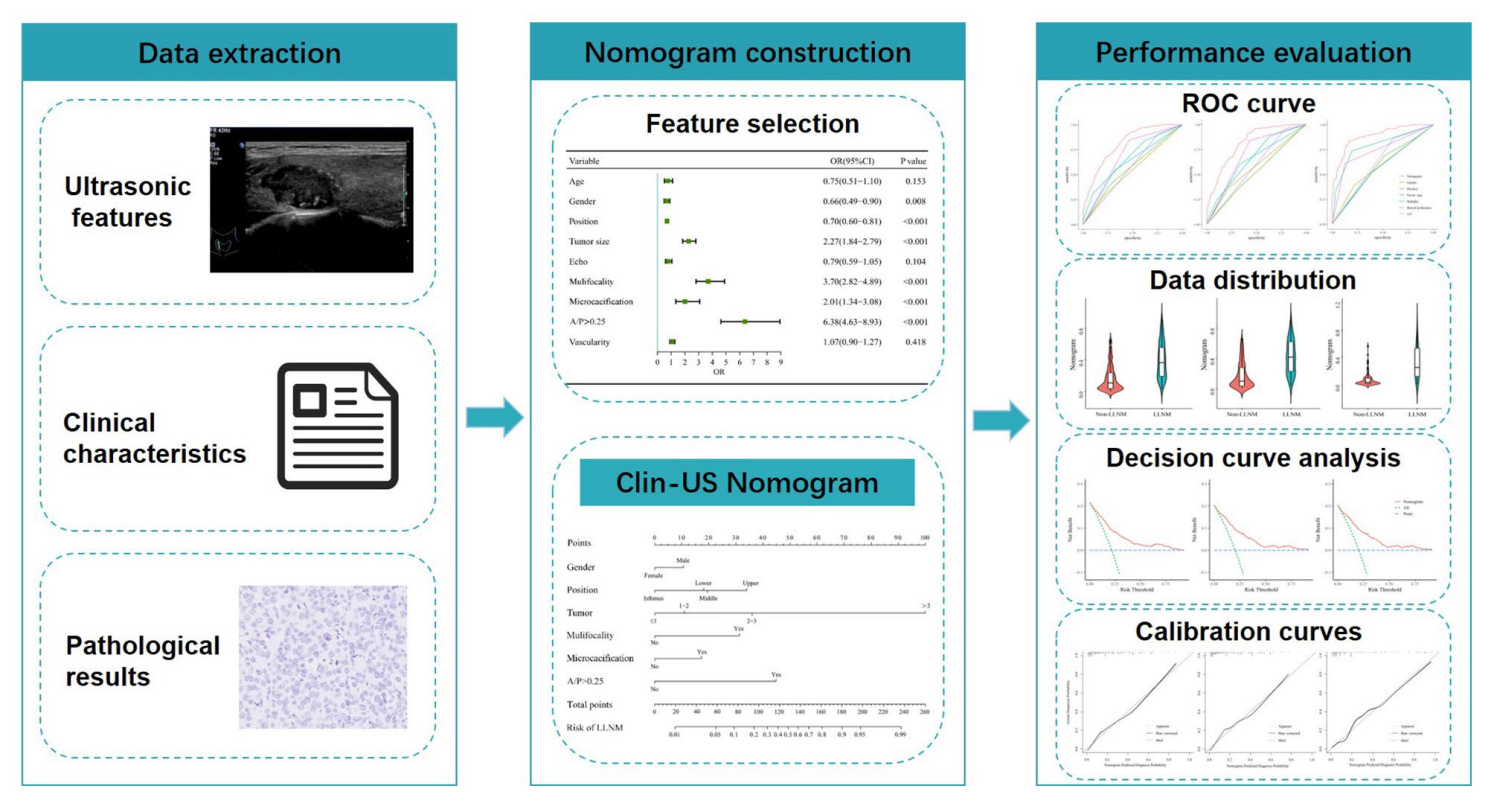
Schematic diagram of the Clin-US nomogram for predicting the risk of LLNM. First, ultrasonic and clinical features were extracted, and logistic analysis was performed according to pathological results. Second, features with significant differences were selected and the nomogram was constructed. Last, the predicting performance of Clin-US nomogram was evaluated by ROC, decision and calibration curves

The nodules in the primary cohort (hospital 1) were divided randomly into a training cohort and an internal testing cohort with a 7:3 ratio. Student’s independent t-test and χ2 test were applied to select the risk parameters associated with LLNM significantly. Multivariate regression analysis conducted by combining significant clinical and US variables was performed to decide the final indicators for predicting LLNM. Statistical significance was determined by a two- tailed P < 0.05. Based on the multivariate analysis in the training cohort, the Clin-US nomogram was developed. Then, the Clin-US nomogram was then internally validated and externally tested. Fig. 2 is the schematic diagram of constructing and verifying the Clin-US nomogram for predicting the probability of LLNM.

### Predictive performance of the Clin-US nomogram

Receiver operating characteristic (ROC) curve analysis was utilized to evaluate the diagnostic performance of the Clin-US nomogram. The area under the curve (AUC) was used for quantitative measurement and differentiation. The calibration curve and Hosmer-Lemeshow test were applied to judge the correction effect of the Clin-US nomogram on the training and testing cohorts. Decision curve analysis (DCA) was used to evaluate clinical utility of the predictive Clin-US nomogram by calculating the net benefit at disparate threshold probabilities. According to the nomogram algorithm, the predicted probability of each nodule was calculated and defined as Nomoscore. Then, the best cutoff value was determined by maximizing the Youden index. The predictive performance of the optimal cutoff value of the Nomoscore was evaluated by the AUC, sensitivity, and specificity.

### Statistical analyses

The Mann-Whitney U test and chi-square test were separately used to compare the differences in continuous variables and categorical variables. The model predictions were assessed by sensitivity, specificity, positive predictive value (PPV), negative predictive value (NPV), AUC and 95% confidence interval (CI) as well as calibration curves in both the training and testing cohorts. Delong test was used to compare different AUC. Calibration plot analysis was performed by bootstrapping with 1,000 replications. All analyses were performed using R statistical software (version 3.3.3; www.R-project.org). P < 0.05 was considered statistically significant.

## Results

### Baseline characteristics of all patients with PTC in three cohorts

In total, 564/2612 (21.6%) and 2048/2612 (78.4%) patients with LLNM and without LLNM were enrolled, respectively. The demographics and sonographic features of the patients were demonstrated in Table 1. The mean age of patients was 44.12 ± 11.17 years for the training cohort, 43.91 ± 11.12 years for the internal testing cohort and 46.56 ± 11.78 years for the external testing cohort. The rate of LLNM in three cohorts was 21.6% (374/1732), 20.4% (118/578) and 23.8% (72/302), respectively.

**Table 1:** Patient characteristics of the training and testing cohorts

Characteristic	Training Cohort (% )	Internal Testing Cohort (%)	External Testing Cohort (% )
*n*	1732	578	302
**Age**, mean ± SD, years	44.12 ± 11.17	43.91 ± 11.22	46.56 ± 11.78
**Gender, n**			
Female	1334 (77.0)	435 (75.3)	224 (74.2)
Male	398 (23.0)	143 (24.7)	78 (25.8)
**Tumor size (cm)**	1.45 ± 0.85	1.42 ± 0.75	1.08 ± 0.78
**Tumor location**			
Left lobe	798 (46.1)	249 (43.1)	152 (50.3)
Right lobe	880 (50.8)	313 (54.1)	143 (47.4)
Isthmus	54 (3.1)	16 (2.8)	7 (2.3)
**Tumor position**			
Upper pole	528 (30.5)	186 (32.2)	36 (11.9)
Middle pole	432 (24.9)	127 (22.0)	134 (44.4)
Lower pole	722 (41.7)	265 (45.8)	132 (43.7)
**Composition**			
Mixed cystic and solid	16 (0.9)	8 (1.4)	9 (3.0)
Solid	1716 (99.1)	570 (98.6)	293 (97.0)
**Internal echo**			
Hyper or isoechoic	25 (1.4)	10 (1.7)	6(1.9)
Hypoechoic	1184 (68.4)	398 (68.9)	289(95.6)
Markedly hypoechoic	523 (30.2)	170 (29.4)	7(2.3)
**Margins**			
Well-defined	127 (7.3)	32 (5.5)	48 (15.9)
Ill-defined	1605 (92.7)	546 (94.5)	254 (84.1)
**Shape**			
A/T < 1	423 (24.4)	125 (21.6)	111 (36.8)
A/T ≥ 1	1309 (75.6)	453 (78.4)	191 (63.2)
**Microcalcification**			
No	361 (20.8)	102 (17.6)	101 (33.4)
Yes	1371 (79.2)	476 (82.4)	201 (66.6)
**Vascularization (blood flow)**			
0	1193 (68.9)	410 (70.9)	5 (1.7)
1	330 (19.0)	115 (19.9)	197 (65.2)
2	157 (9.1)	40 (6.9)	65 (21.5)
3	52 (3.0)	13 (2.3)	35 (11.6)
**Abutment/perimeter**			
≤ 0.25	789 (45.6)	280 (48.4)	229 (75.8)
> 0.25	943 (54.4)	298 (51.6)	73 (24.2)
**Mulifocality**			
No	1102 (63.6)	355 (61.4)	176 (58.3)
Yes	630 (36.4)	223 (38.6)	126 (41.7)
**Hashimoto thyroiditis**			
Negative	1335 (77.1)	447 (77.3)	263 (87.1)
Positive	397 (22.9)	131 (22.7)	39 (12.9)
**BRAF V600E mutation**			
No	246 (14.2)	70 (12.1)	NA
Yes	1486 (85.8)	508 (87.9)	NA
**LLNM status in pathology**			
Non-LLNM	1358 (78.4)	460 (79.6)	230 (76.2)
LLNM	374 (21.6)	118 (20.4)	72 (23.8)

Note—A/T < 1, wider-than-tall; A/T ≥ 1, taller than wide; LLNM, lateral lymph node metastases

### Constructing and evaluating nomogram

**Fig. 3:**
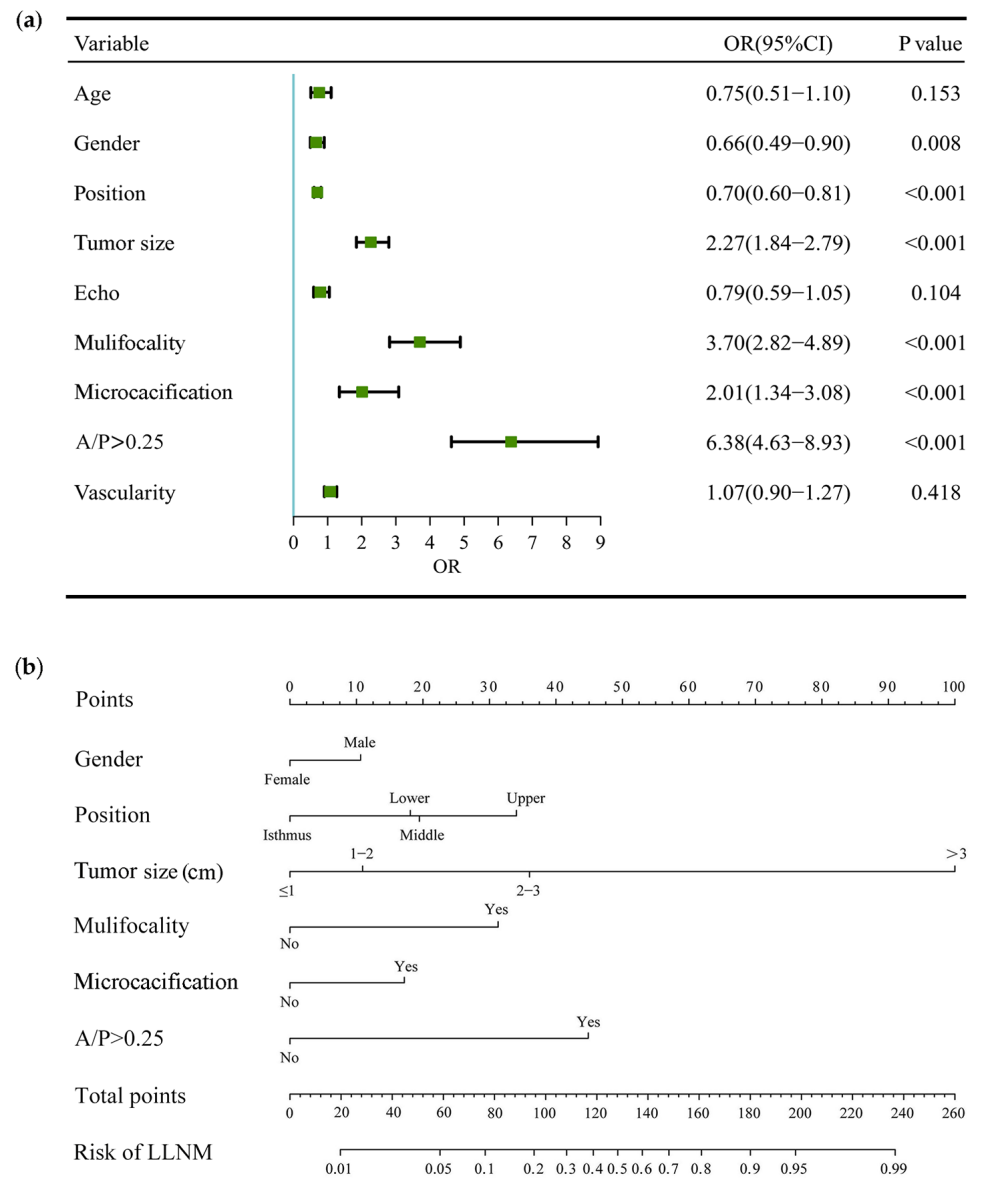
Forest plot of risk factors and the Clin-US nomogram for estimating the risk of LLNM in PTC. (**a**) Forest plot of risk factors in multivariable logistic regression analysis for LLNM. (**b**) The proposed nomogram based on preoperative data for assessing the risk of LLNM in PTC patients. OR, odds ratio; LLNM, lateral lymph node metastasis; CI, confidence interval

In the training cohort, nine predictors were significantly different in the LLNM and non-LLNM groups by univariate analysis, which were age, gender, tumor size, tumor position, internal echo, microcalcification, vascularization, A/T and mulifocality (Table 2). Then, multivariate regression analysis was applied to construct a Clin-US nomogram for predicting LLNM based on these nine risk predictors. Multivariate logistics regression analysis identified tumor position, gender (male), microcalcification, tumor size, mulifocality, and A/P > 0.25 as independent predictive risk factors for LLNM (Table 3; Fig. 3a). The Clin-US nomogram was established based on these six indicators (Fig. 3b). The nomogram scored 11 for male, 18 for lower, 19 for middle, 34 for upper, 11 for 1-2 cm in size, 36 for 2-3 cm in size, 100 for size > 3 cm, 31 for multifocal, 17 for microcalcification, 45 for A/P > 0.25. The final nomoscore cutoff for positivity is 108 with the corresponding probability of LLNM is 0.30.

**Table 2:** Patient characteristics of the PTC with LLNM and PTC without LLNM groups in the training cohort

Characteristic	Non-LLNM group (% )	LLNM group (% )	*p*
*n*	1358	374	
**Age** (years)			**0.016**
≤ 55	1126 (82.9)	330 (88.2)	
>55	232 (17.1)	44 (11.8)	
**Gender, n**			**0.0028**
Female	1068 (78.6)	266 (71.1)	
Male	290 (21.4)	108 (28.9)	
**Tumor size (cm)**			**<0.0001**
c	515 (37.9)	60 (16.0)	
>1 and ≤ 2	709 (52.2)	199 (53.2)	
>2 and ≤ 3	133 (9.8)	99 (26.5)	
>3	1 (0.1)	16 (4.3)	
**Tumor position**			**<0.0001**
Upper pole	377 (27.8)	151 (40.4)	
Middle pole	350 (25.8)	82 (21.9)	
Lower pole	588 (43.3)	134 (35.8)	
Isthmus	43 (3.1)	7 (1.9)	
**Composition**			0.0631
Mixed cystic and solid	9 (0.7)	7 (1.9)	
Solid	1349 (99.3)	367 (98.1)	
**Internal echo**			**0.0011**
Hyper or isoechoic	17 (1.3%)	8 (2.2)	
Hypoechoic	903 (66.5)	281 (75.1)	
Markedly hypoechoic	438 (32.2)	85 (22.7)	
**Margins**			0.836
Well-defined	101 (7.4)	26 (7.0)	
Ill-defined	1257 (92.6)	348 (93.0)	
**Shape**			0.0737
A/T < 1	318 (23.4)	105 (28.1)	
A/T ≥ 1	1040 (76.6)	269 (71.9)	
**Microcalcification**			**<0.0001**
No	327 (24.1)	34 (9.1)	
Yes	1031 (75.9)	340 (90.9)	
**Vascularization (blood flow)**			**0.0002**
0	964 (71.0)	229 (61.2)	
1	244 (18.0)	86 (23.0)	
2	106 (7.8)	51 (13.6)	
3	44 (3.2)	8 (2.2)	
**Abutment/perimeter**			**<0.0001**
≤ 0.25	731 (53.8)	58 (15.5)	
> 0.25	627 (46.2)	316 (84.5)	
**Mulifocality**			**<0.0001**
No	930 (68.5)	172 (46.0)	
Yes	428 (31.5)	202 (54.0)	
**Hashimoto thyroiditis**			0.2229
Negative	1056 (77.8)	279 (74.6)	
Positive	302 (22.2)	95 (25.4)	
**BRAF V600E mutation**			0.0570
No	181 (13.3)	65 (17.4)	
Yes	1177 (86.7)	309 (82.6)	

Note—LLNM, lateral lymph node metastases; A/T < 1, wider-than-tall; A/T ≥ 1, taller than wide

**Table 3:** Risk factors for lateral cervical lymph node metastasis in the training cohort

Clinical variable	Multivariate analysis				
	0R	Estimate	Std. Error	z value	*p* value
Age	0.75	-0.28202	0.19737	-1.429	0.153
Gender	0.66	-0.40949	0.15382	-2.662	**0.0078**
Position	0.70	-0.36106	0.07399	-4.880	**<0.0001**
Tumor size	2.27	0.81763	0.10585	7.724	**<0.0001**
Internal echo	0.79	-0.23818	0.14645	-1.626	0.1039
Mulifocality	3.70	1.30892	0.14050	9.316	**<0.0001**
Mircocacification	2.01	0.69883	0.21115	3.31	**0.0009**
A/P > 0.25	6.38	1.85282	0.16748	11.063	**<0.0001**
Vascularization	1.07	0.07006	0.08646	0.81	0.417805

Note—OR, odds ratio; A/P, Abutment/perimeter

### Predictive performance of Clin-US nomogram

**Fig. 4:**
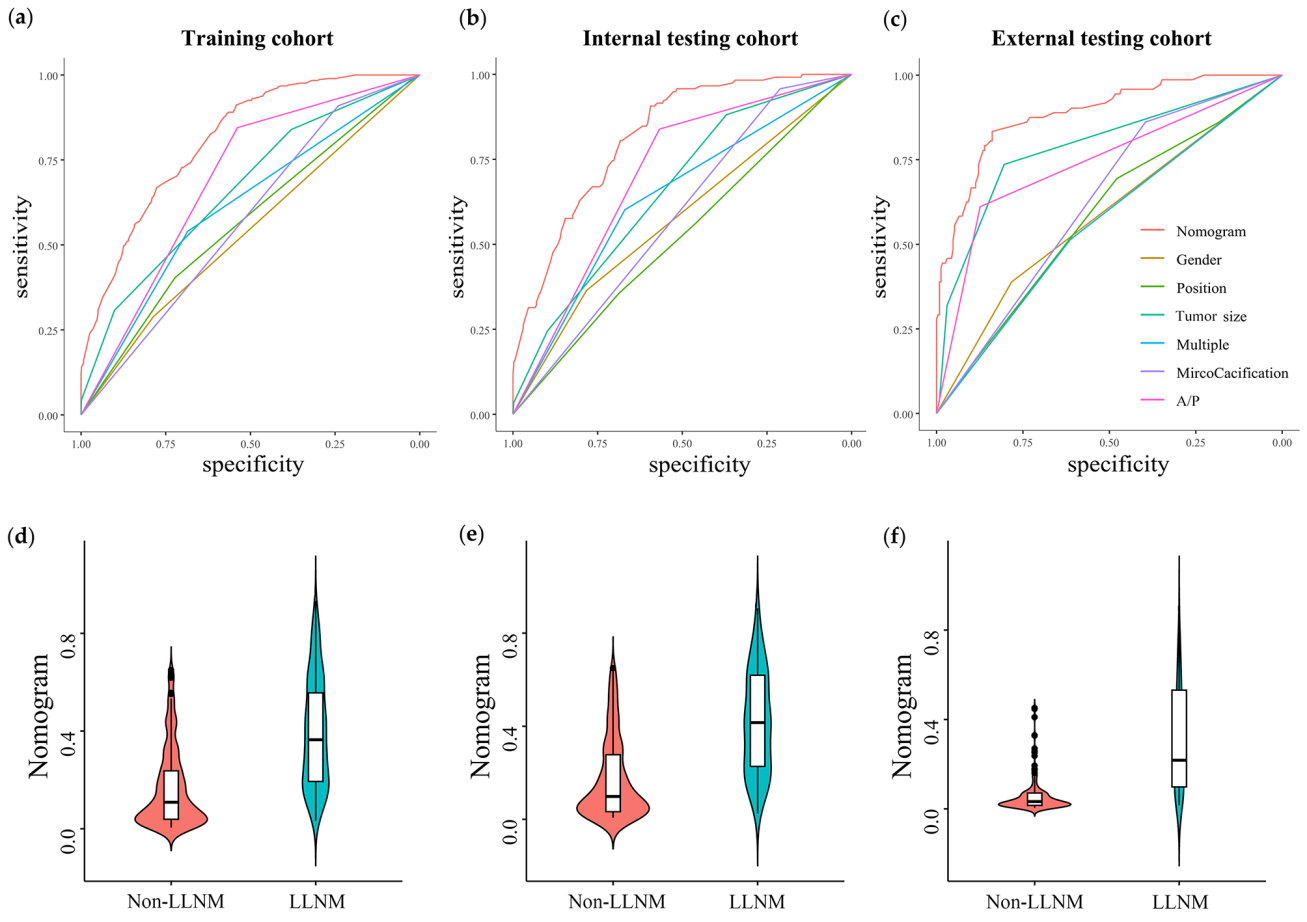
Predictive performance of the Clin-US nomogram and US features in discrimination of LLNM and non-LLNM in three cohorts. ROC curves of Clin-US nomogram compared to ultrasonic features in the training cohort (**a**), internal testing cohort (**b**) and external testing cohort (**c**). The violin plot shows the data distribution, including a box plot (**d-f**)

The performance of the Clin-US nomogram in predicting LLNM is shown in Table 4, Table S1 and Fig. 4. The AUC of the Clin-US nomogram in the training cohort was 0.813 (95% CI, 0.790–0.835), 0.815 (95% CI, 0.775–0.854) in the internal cohort, and 0.870 (95% CI, 0.822–0.917) in the external test cohort. Both of ROC curves and violin plots in Fig. 4 revealed that the Clin-US nomogram showed excellent prediction ability in three cohorts. The Clin-US nomogram achieved a highest AUC in the training cohort, with an accuracy of 77.89%, a specificity of 83.58% and a sensitivity of 57.22%. Similarly, the AUC of the Clin-US nomogram was higher than other six independent risk factors in the both internal and external testing cohort, with a highest sensitivity of 89.47% in the external testing cohort. The cross-cross matrices of Clin-US nomogram in three cohorts are shown in Fig. S1.

**Table 4:** Predictive performance of the different models for the training and testing cohorts

	AUC (95% CI)	Sensitivity (%)	Specificity (%)
Training cohort			
Clin-US Nomogram	0.813 (0.790–0.835)	57.22	83.58
Sex	0.538 (0.512–0.563)	78.65	28.88
Position	0.567 (0.536–0.598)	72.24	40.37
Tumor size	0.665 (0.632–0.694)	37.92	83.96
Multifocality	0.613 (0.584–0.641)	68.48	54.01
Mircocacification	0.575 (0.556–0.593)	24.08	90.91
A/P>0.25	0.692 (0.669–0.714)	53.83	84.49
**Internal testing cohort**			
Clin-US Nomogram	0.815 (0.775–0.854)	58.47	81.52
Sex	0.574 (0.526–0.621)	78.26	36.44
Position	0.522 (0.467–0.577)	68.70	35.59
Tumor size	0.659 (0.613–0.705)	36.96	88.14
Multifocality	0.636 (0.586–0.685)	66.96	60.17
Mircocacification	0.584 (0.558–0.610)	21.09	95.76
A/P>0.25	0.703 (0.663–0.744)	56.74	83.90
**External testing cohort**			
Clin-US Nomogram	0.870 (0.822–0.917)	89.47	80.57
Sex	0.586 (0.523–0.648)	78.26	38.89
Position	0.583 (0.513–0.654)	47.83	69.44
Tumor size	0.789 (0.730–0.849)	80.43	73.61
Multifocality	0.564 (0.497–0.630)	61.30	51.39
Mircocacification	0.628 (0.577–0.680)	39.57	86.11
A/P>0.25	0.743 (0.682–0.803)	87.39	61.11

Note—AUC, area under the curve; A/P, Abutment/perimeter; CI, confidence interval

**Fig. 5:**
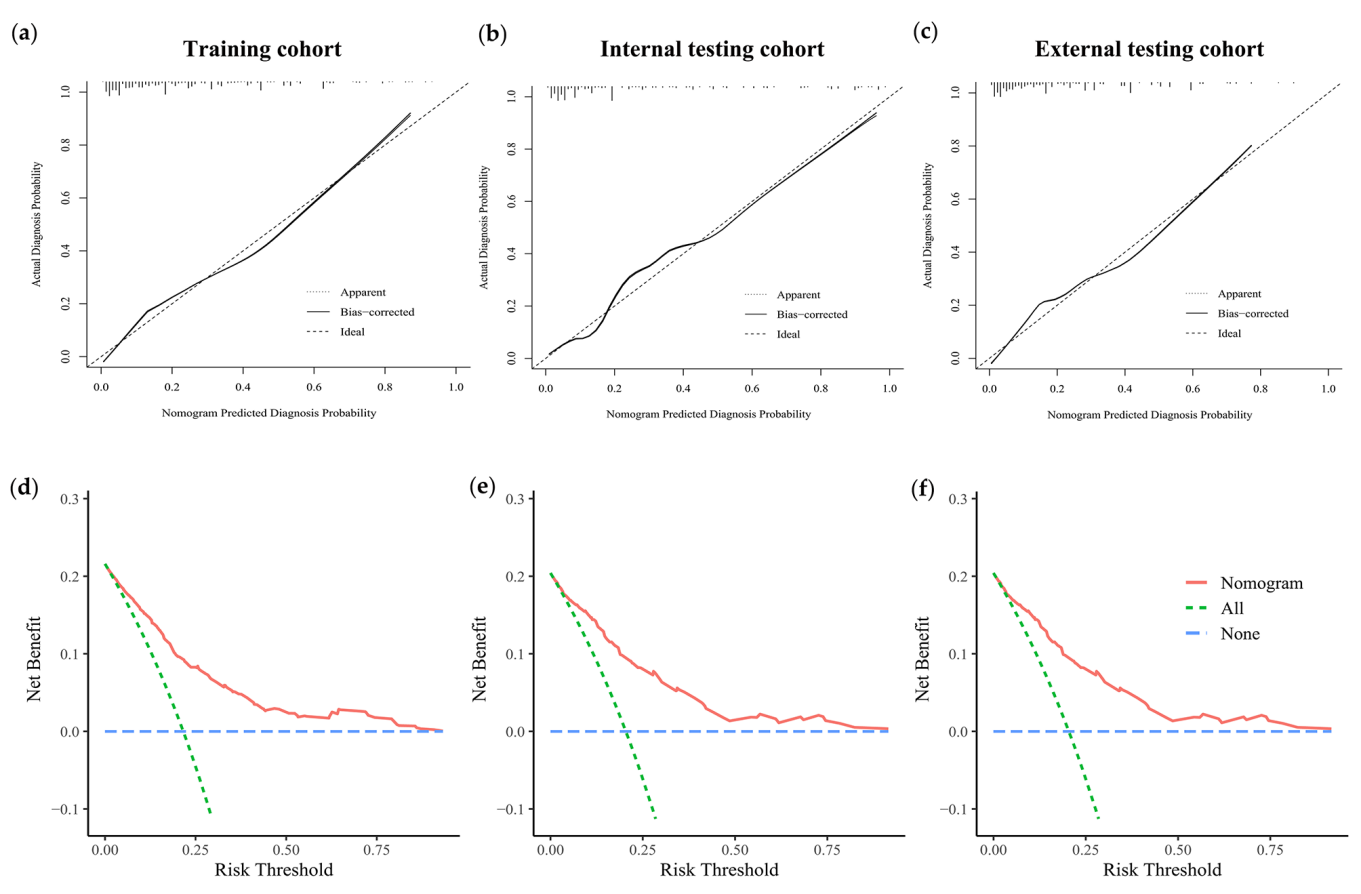
Calibration curves and decision curve analysis. (**a-c**) Calibration curve of the Clin-US nomogram in the training cohort and two validation cohorts. (**d-f**) Decision curve analysis of the Clin-US nomogram in the training cohort and two validation cohorts. The x-axis represents the threshold probability, and the y-axis represents the net benefit

The calibration curves of Clin-US nomogram exhibited good consistency between the bias-corrected prediction and ideal reference lines with an additional 1000 bootstraps in the training and two testing cohorts (Fig. 5a-c). We also performed decision curve analysis (DCA) to compare the clinical availability and benefits of Clin-US nomogram and traditional US methods in estimating the risk of LLNM. The DCA curves of the Clin-US nomogram showed greater net benefits across a range of LLNMs risks in the three cohorts than the other factors (Fig. 5d-f).

### Clinical application of the Clin-US nomogram

**Fig. 6:**
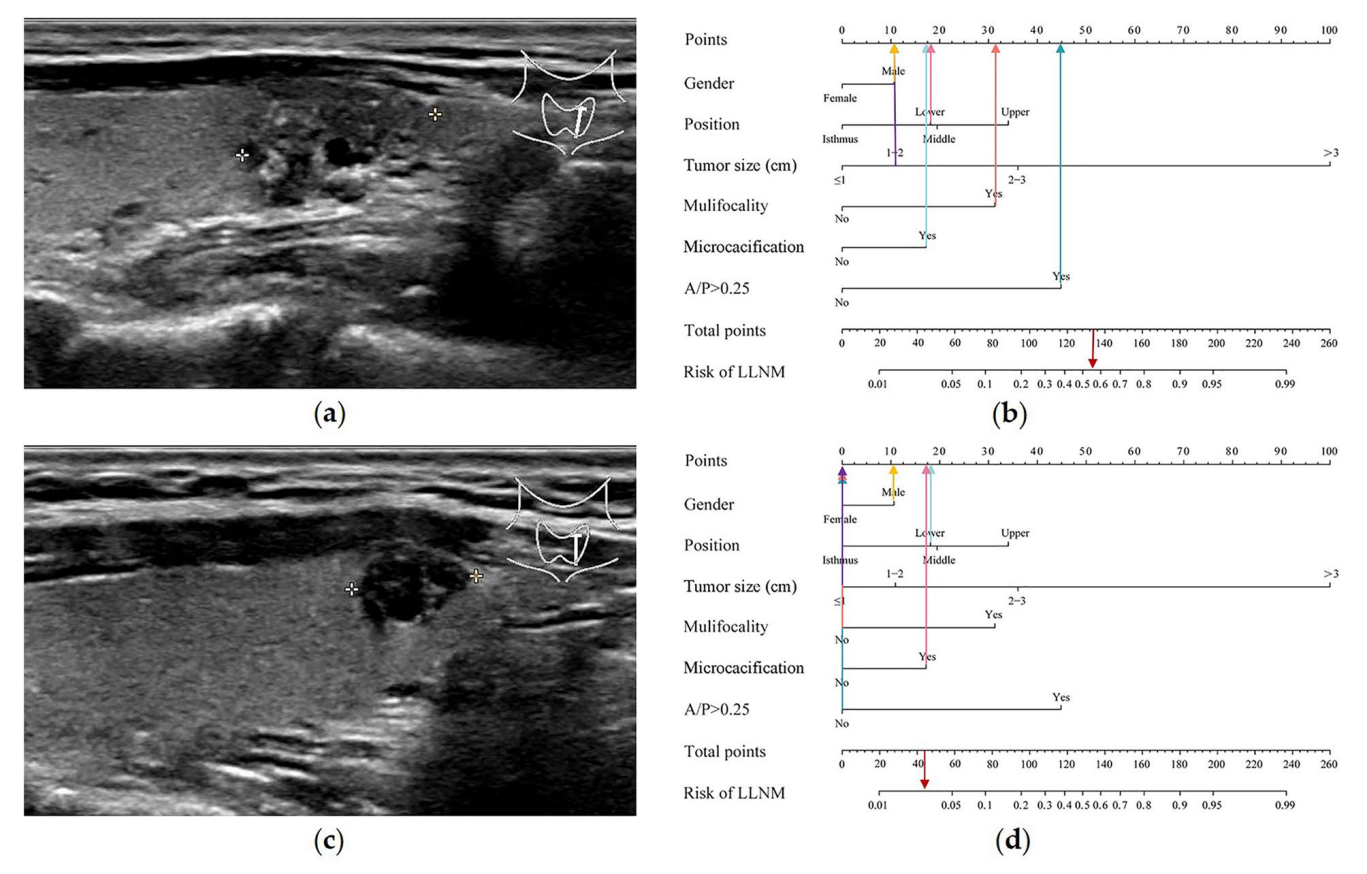
Examples of clinical application of the Clin-US nomogram. (**a**) Image was obtained from a 22-year-old man with nodule in the left thyroid. (**b**) The nomogram resulted in a total score of 133 points for man (11 points), lower pole (18 points), max diameter of 1.43 cm (11 points), mulifocality (31 points), microcacification (17 points), A/P > 0.25 (45 points). The corresponding risk of LLNM was 0.55, and the pathological result of the nodule was PTC with LLNM. (**c**) Image was obtained from a 39-year-old man with nodule in the left thyroid. (**d**) The total points of the nomogram were 46 for man (11 points), lower pole (18 points), max diameter of 0.91 cm (0 points), unifocal (0 points), microcacification (17 points), and A/P < 0.25 (0 points). The corresponding risk of LLNM was low (< 0.05), and the pathological result of the nodule was PTC without LLNM.

Representative examples of predicting the risk of LLNM in PTC patients are shown in Fig. 6. The thyroid nodule in Fig. 6a was obtained from a man with a tumor on the lower pole of the left thyroid, who had three high-risk sonographic features (microcalcification, mulifocality, and A/P > 0.25). The maximum diameter of this tumor is 1.43 cm. The probability of LLNM using the nomogram model was 55% (Fig. 6b). The tumor was PTC with LLNM of levels II, III, IV and VI according to postoperative pathological report. The male patient 2 with a nodule on the lower pole of the left thyroid in Fig. 6c, who had only one high-risk sonographic feature (microcalcification). The maximum diameter of this tumor is 0.91 cm. The probability of LLNM using the nomogram model was less than 5% (Fig. 6d). The nodule was PTC without LLNM according to postoperative pathological report and FNA-TG test.

## Discussion

In this research, we constructed and validated the Clin-US nomogram based on clinical and ultrasound characteristics to predict the probability of LLNM in patients with PTC. The Clin-US nomogram effectively categorized patients based on their risk of LLNM, and achieved excellent performance in both internal and external testing cohorts. Therefore, the preoperative probability of LLNM can be estimated individually and noninvasively. Our study has several advantages: (I) To the best of our knowledge, this is the largest scale retrospective consecutive multicenter study to construct and evaluate a nomogram to predict status of LLNM in PTC. (II) Different from published studies based on radiomics [14], contrast enhanced ultrasound (CEUS) [15], or elastography [16], this novel nomogram only incorporated clinical and gray-scale US factors, which increasing the general applicability of the model. It was particularly important for PTC patients in underdeveloped countries and regions. (III) Comparing with other machine learning (ML) models in previous studies [2, 17], the Clin-US nomogram we proposed had a better interpretability and maneuverability in clinical practice with a similar diagnostic performance.

Precise preoperative checking to determine status of lateral lymph nodes is essential for clinicians. Although many previous studies have explored the risk factors affecting LLNM of PTC, the results have not always been consistent. We confirmed that suspicious US features of A/P > 0.25 and microcalcification were independent predictors of LLNM, which was consistent with our previous study [13]. Capsular extension, especially the degree of capsular extension and disruption, can predict extrathyroidal extension and invasion in many researches. Ye et al. indicated capsular extension > 50% in the LLNM group of PTC was the most common, comparing with no LNM group and central LNM group [18], which was similar to our findings. Microcalcification could reflect the psammoma bodies in pathology, which was a result of necrosis and calcification of cancer cells and was a specific indicator for PTC diagnosis. It was also reported associated with lymph node metastasis significantly [19].

In this study, the clinical characters of gender (male), tumor maximum diameter, mulifocality, and position of the lesion also showed significant significance contributing to LLNM. Similarly, Feng et al. considered that LLNM was independently related to tumor size, the number of foci, and location. Zhuo et al. [10] identified male sex, tumor size, thyroid nodules, irregular tumor shape, rich lymph node vascularity and location of lymph node as independent risk factors for LLNM. Numerous previous studies have identified mulifocality as a risk predictor for LNM. Wang et al. considered that mulifocality was an independent risk factors for both central lymph node metastasis (CLNM) and LLNM [20].

To our surprise, BRAF V600E mutation had no significant difference in univariate analysis. The BRAFV600E gene was reported to be an important biomarker for the progression of PTC [21]. On the contrary, Liu et al. [22] indicated that absence of BRAF V600E mutation was more prone to LLNM. A taller than wide (A/T) shape is an insensitive but highly specific indicator of malignant thyroid tumor [11]. But it had no significant correlation with LLNM in univariate analysis in our findings. Previous reports have suggested that PTC patients with Hashimoto’s thyroiditis were associated with less aggressive diseases [20]. In other words, absence of Hashimoto’s thyroiditis is independent clinical feature of PTC patients who have cervical lymph nodes metastasis [23]. However, Hashimoto’s thyroiditis had no significant difference in univariate analysis in this study. The possible reason is that we judged the presence or not of Hashimoto’s thyroiditis by preoperative US but not postoperative pathology. In addition, although vascularization was statistically significant in univariate analysis, it was not significant in multivariate analysis. One possible reason is that the assessment to distribution of internal blood vessels by US is unreliable and easily influenced by the operators and the machines.

There are many studies using ML to predict risk of LNM or CLNM in PTC patients [24–26], but only a few ML models were applied to LLNM. Although many models showed good performance, they were not convenient for clinical application since the predicted probability could not be obtained intuitively. Comparing to other ML models, the Clin-US nomogram is a reliable and easy-to-use clinical prediction model with better interpretability and maneuverability.

A few previous studies had constructed nomograms to estimate LLNM based on different risk parameters for LLNM of PTC. Jin et al. established a nomogram for estimating LLNM with clinicopathologic factors, such as Hashimoto thyroiditis, numbers of tumor, serum thyroid-stimulating hormone level, and metastatic rate of central lymph nodes [27]. Wang et al. created a nomogram to predict level V lymph node metastasis incorporating extra nodal extension, unilateral central lymph node metastasis, level II-IV metastasis, and lymph node size [28]. Unfortunately, some of these risk factors cannot be beneficial for preoperative prediction as some parameters used in these assessment models included postoperative pathologic features which were not obtainable before operation. Besides, multimodal imaging data including CEUS, ultrasound elastography, and three-dimensional imaging are not available in underdeveloped areas with little medical resources. Some researchers developed nomogram based on ultrasonography radiomics to predict the LLNM in PTC patients [14, 29]. However, US radiomic features are often very abstract, unspecific, and difficult to apply directly. In addition, some studies predict risk of lymph node metastasis based on sonographic features of lateral lymph nodes [10]. But occult LLNM may occur and not be detected by preoperative regular US [30], which led to that the lymph nodes could not be evaluated by US. The Clin-US nomogram we constructed is based on available clinical and US features of primary thyroid tumor and thus can be helpful for preoperative decision making, especially for PTC patients in underdeveloped countries and regions.

This study has some limitations. First, it was designed retrospectively, and the assessment of static US images has an inherent limitation to the precision of US interpretation. Further improvements will be needed through large-scale prospective studies. Second, US scanning and biopsy were conducted by different doctors using differing US machines. Therefore, the determination of US categorization and biopsy may have been affected by operator experience. Lastly, given that LND was only performed in patients with high suspicion of LLNM based on preoperative imaging and FNAC, microscopic metastases might be overlooked.

## Conclusions

In conclusion, the Clin-US nomogram could effectively predict the probability of LLNM in patients with PTC preoperatively according to the suspicious US and clinical characters. We identified gender (male), maximum diameter of tumor, mulifocality, position, micro-calcification, and A/P as the most influentials in predicting LLNM. The proposed nomogram will be useful for estimating lateral neck lymph nodes and guiding the clinical diagnosis and treatment process of patients with PTC.

## Electronic supplementary material

Below is the link to the electronic supplementary material.

Supplementary Material 1

Supplementary Material 2

## Data Availability

The datasets used during the current study are available from the corresponding author on reasonable request.

## Declarations

**Ethics approval and consent to participate**.

This research project was approved by the Ethics Committee of Tianjin Medical University Cancer Institute and Hospital. Patient consent was waived due to the retrospective nature of the study.

**Consent for publication**.

Publication was approved by all authors and tacitly or explicitly by the responsible authorities where the work was carried out.

## Abbreviations

AUC: Area under curve
A/P: Abutment/perimeter
A/T>1: Taller than wide
CEUS: Contrast enhanced ultrasound
CI: Confidence interval
Clin-US: Clinical-ultrasound
CLNM: Central lymph node metastasis
CND: Central neck dissection
DCA: Decision curve analysis
FNAC: Fine needle aspiration cytology
LLNM: Lateral lymph node metastases
LND: Lateral neck dissection
LNM: Lymph node metastasis
ML: Machine learning
NPV: Negative predictive value
PPV: Positive predictive value
PTC: Papillary thyroid carcinoma
ROC: Receiver operating characteristic
US: Ultrasound
