# Supplementary figures and images for: Nomogram for preoperative estimation risk of lateral cervical lymph node metastasis in papillary thyroid carcinoma: a multicenter study
Source: Cancer Imaging. 2023 Jun 1;23:55. doi: 10.1186/s40644-023-00568-5 (PMC10236734; doi:10.1186/s40644-023-00568-5)

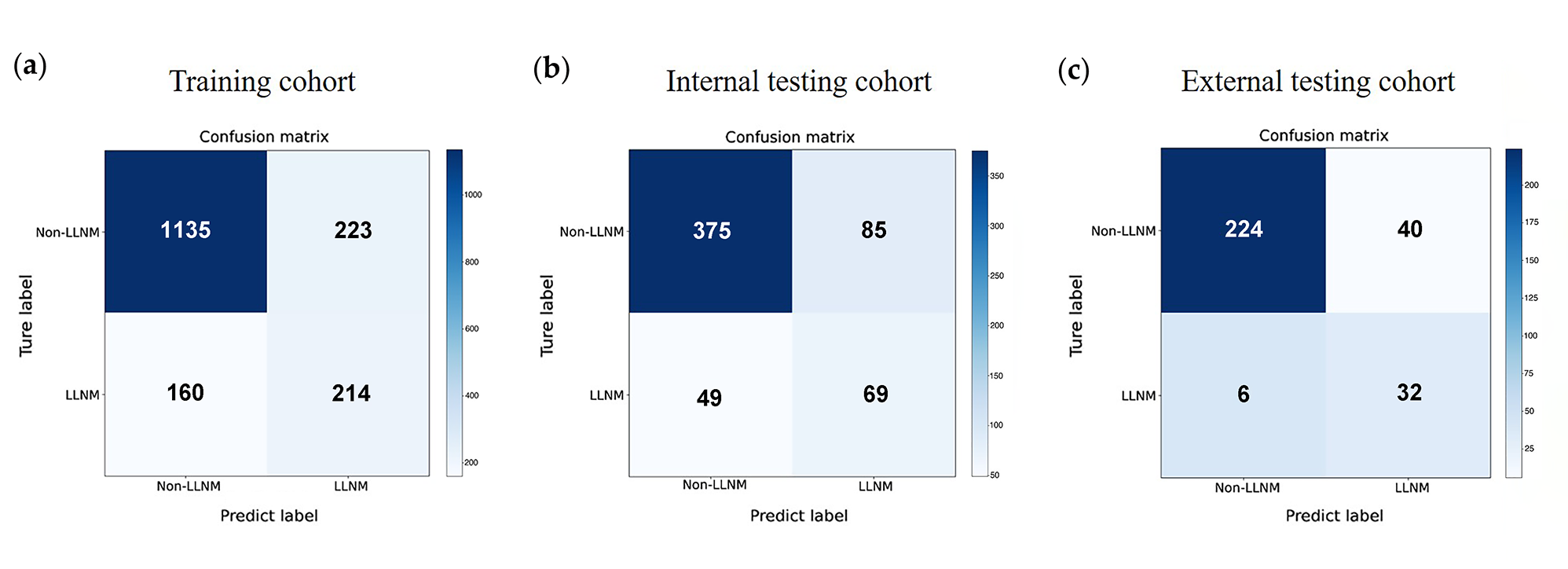

Supplement: Supplementary file 1 — Supplementary Material 1 [file 40644_2023_568_MOESM1_ESM.tif]
