# Supplementary material for: Nomogram for preoperative estimation risk of lateral cervical lymph node metastasis in papillary thyroid carcinoma: a multicenter study
Source: Cancer Imaging. 2023 Jun 1;23:55. doi: 10.1186/s40644-023-00568-5 (PMC10236734; doi:10.1186/s40644-023-00568-5)
Supplement: Supplementary file 2 — Supplementary Material 2 [file 40644_2023_568_MOESM2_ESM.docx]

|  | **AUC**  **(95% CI)** | **Accuracy**  **(95% CI)** | **Specificity**  **(%)** | **Sensitivity**  **(%)** | **NPV**  **(%)** | **PPV**  **(%)** |
| --- | --- | --- | --- | --- | --- | --- |
| **Training cohort** | 0.8126  (0.7902-0.8350) | 0.7789 (0.7586, 0.7982) | 83.58 | 57.22 | 87.64 | 48.97 |
| **Internal testing cohort** | 0.8145  (0.7750-0.8540) | 0.7682  (0.7316, 0.8020) | 81.52 | 58.47 | 88.44 | 44.81 |
| **External testing cohort** | 0.8695  (0.8218-0.9172) | 0.8477  (0.8021, 0.8863) | 80.57 | 89.47 | 97.39 | 44.44 |

Table S1. Predictive performance of the Clin-US nomogram for the training and testing cohorts.

Note—CI, confidence interval; PPV, positive predictive value; NPV, Negative predictive value
